# Considerations for using amyloid-targeting therapies for the treatment of early symptomatic Alzheimer’s disease

**DOI:** 10.3389/fneur.2026.1863897

**Published:** 2026-08-10

**Authors:** Andrew E. Budson, Peter A. Ljubenkov, Aaron Ritter, Cara Leahy, Vijay K. Ramanan, Jared Brosch, Anna Burke, Brandy R. Matthews

**Affiliations:** 1 Neurology Service, Veterans Affairs Boston Healthcare System, Boston, MA, United States; 2 Alzheimer’s Disease Research Center, Boston University Chobanian and Avedisian School of Medicine, Boston, MA, United States; 3 Department of Neurology, Memory and Aging Center, University of California San Francisco, San Francisco, CA, United States; 4 Hoag Medical Group, Irvine, CA, United States; 5 Memorial Healthcare, Owosso, MI, United States; 6 Department of Neurology, Mayo Clinic, Rochester, MN, United States; 7 Department of Neurology, Indiana University, Indianapolis, IN, United States; 8 Barrow Neurological Institute, Phoenix, AZ, United States; 9 Eli Lilly and Company, Indianapolis, IN, United States

**Keywords:** Alzheimer’s disease, amyloid, clinical neurology, dementia, geriatrics

## Abstract

Amyloid-targeting therapies (ATTs), including donanemab and lecanemab, are FDA-approved disease-modifying therapies for Alzheimer’s disease in the mild cognitive impairment or mild dementia stages. Early initiation is important for optimal outcomes. This article summarizes practical strategies for implementing ATTs in clinical practice, focusing on leadership alignment and infrastructure development, as well as patient identification, diagnosis, and monitoring protocols. Key recommendations include establishing appropriate patient tracking systems, infusion and imaging protocols, and interprofessional collaboration. Approaches for systematic patient routing (e.g., hub-and-spoke models, fast-track mechanisms) and biomarker-integrated assessments are critical to facilitating timely diagnosis and treatment. Education for patients, care partners, and clinicians is crucial for managing the risks associated with amyloid-related imaging abnormalities. Successful ATT implementation requires coordinated systems, timely diagnosis, and robust safety monitoring. These measures can enable frameworks for ATT administration to optimize outcomes for patients, care partners, and health systems.

## 1 Introduction

Two amyloid-targeting therapies (ATTs) approved by the United States (US) Food and Drug Administration (FDA) are being used for the treatment of patients with Alzheimer’s disease (AD) in the mild cognitive impairment and mild dementia stages. Donanemab and lecanemab have demonstrated slowing of cognitive and functional decline across trials, subgroups, and outcome measures over 18 months, with early evidence for persistent benefit thereafter in open-label and blinded extension studies (1–3). As the first widely-utilized disease-modifying therapies for the treatment of AD, the real-world knowledge and infrastructure for identifying appropriate patients and optimizing drug delivery are being developed across practices that care for patients with AD (4–7).

Because initiation of treatment in the earlier symptomatic stages of AD is an important factor in better outcomes (1–3, 8), clinics and health systems need to facilitate early and accurate diagnosis and access to appropriate treatment options. Here, we provide practical guidance to support the safe and effective implementation of ATTs in clinical practice. Based on recent experience in the US healthcare system, this guidance is primarily intended for neurologists, geriatricians, psychiatrists, and healthcare teams who care for patients with AD. Although these specialists are currently central to ATT prescribing and safety monitoring, we also acknowledge that the specialist workforce is insufficient to meet demand (9, 10) and highlight that primary care clinicians participating in shared-care models are central, rather than peripheral, to feasible implementation.

## 2 Practical examples

Before implementing ATTs, we strongly recommend that US-based clinical practices compile their own toolbox of publicly available resources. Patient handouts and provider resources are available on the manufacturers’ websites for donanemab^1^ and lecanemab^2^. Similarly, organizations such as the Alzheimer’s Association, the National Institutes of Health, the Davos Alzheimer’s Collaborative, and the Centers for Medicare and Medicaid Services offer many useful tools (e.g., *APOE* genotyping handouts, general educational materials, fact sheets on potential adverse events).

In addition to publicly available resources, the Supplementary material to this article provides several US-specific templates that may be useful as starting points. All supplements should be reviewed and modified to meet practice-specific workflow needs and to preserve independent clinical judgement. The information in each supplement does not supersede or replace current appropriate use recommendations or approved product labeling.

## 3 Leadership and healthcare system alignment

**Figure 1:**
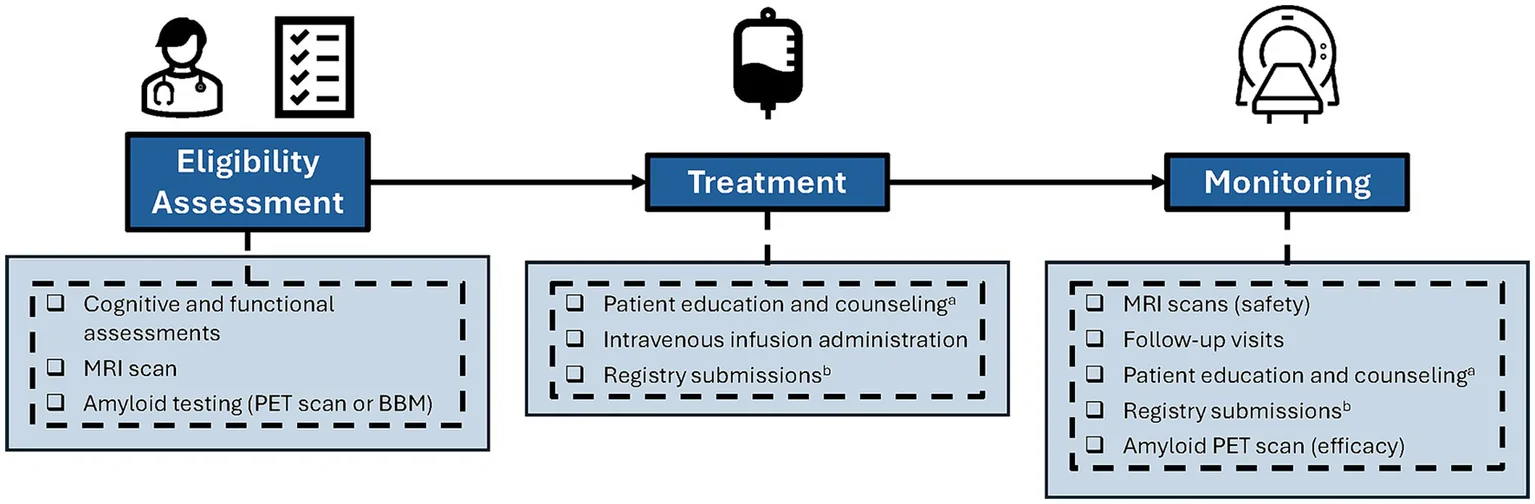
Key patient care touchpoints to support the use of amyloid-targeting therapies (ATTs). Insurance coverage for the listed patient care touchpoints may not be available in all settings or situations. Specific Medicare coding for Current Procedural Terminology (CPT®) is available at CMS.gov. CPT® is a registered trademark of the American Medical Association. Copyright 2024 American Medical Association. All rights reserved. ^a^Patient education and counseling may be billable under Medicare principal care management or chronic care management codes if applicable criteria are met. ^b^Registry submissions are often a condition of reimbursement for ATT administration and not separately billable. BBM, blood biomarkers; MRI, magnetic resonance imaging; PET, positron emission tomography.

Leadership discussions should consider the structural capabilities of the health system, including coverage requirements from the Centers for Medicare and Medicaid Services and other insurance providers (6). For eligible patients, Medicare Part B may cover FDA-approved amyloid-targeting monoclonal antibodies when treatment is furnished in accordance with Centers for Medicare and Medicaid Services coverage requirements, including participation in a qualifying study or registry (11). Key patient touchpoints beyond office visits and ATT administration should be considered to support the sustainable use of ATTs. These touchpoints include patient education, access, and potential revenue from imaging and ongoing patient monitoring (Figure 1). Practices must understand what services are currently billable, including whether infusion centers and imaging facilities are within the same health system. Practices may find it helpful to outline the entire care team, individual roles, and training plans. Lastly, leadership discussions should consider a growth plan with a realistic understanding of how many patients can be managed with a given resource set.

Systematic changes may be needed following leadership alignment. Given the risk of amyloid-related imaging abnormalities (ARIA), an adverse event observed across the ATT class with an associated FDA box warning, a method is needed to track important patient milestones throughout their care. Ideally, tracking tools are integrated into the electronic medical record (EMR) for data fidelity and ease of use (e.g., Supplementary file 1). Infrastructure must ensure seamless MRI scheduling and radiology interpretation to identify ARIA and prevent delayed or inappropriate administration of ATTs. If feasible, we recommend considering an ATT order set in the EMR that creates ‘hard stops’ before dosing until MRI reviews are complete. It may also be useful to develop a detailed infusion protocol (e.g., Supplementary file 2) to outline the expected approach for treatment administration and monitoring within a practice setting. Local emergency and neurology departments must also be prepared to identify and manage ARIA events for those on ATTs.

Finally, implementation will differ across care settings. Within integrated systems such as the Veterans Health Administration, formulary processes, registry participation pathways, and patient population characteristics differ from those in community and academic neurology practices; safety-net systems and Federally Qualified Health Centers face distinct access, staffing, and reimbursement constraints. Practices in these settings should consult system-specific policy guidance and adapt the accompanying templates accordingly.

## 4 Patient identification, diagnosis, and eligibility

Timely identification and diagnosis of patients is important for effective implementation of ATTs in clinical practice. Unfortunately, there are not enough neurologists and other cognitive specialists to diagnose and treat all the patients with AD in the United States (9, 10). The wait to see a neurologist may exceed 6 months in some regions, by which time the patient may have missed the therapeutic window to achieve the greatest potential benefit from ATTs (12). In operational terms, current labeling and appropriate use recommendations define candidacy as AD in the MCI or mild dementia stages. The median time MCI progresses to moderate AD dementia is 4.9 years (13), with the average time in mild AD dementia stage being 2.2 years (14), and the annual probability of mild to moderate dementia transition being 26.6% (14). Specialty physicians need to partner with primary care practices to identify and diagnose patients most likely to benefit from ATTs. One of several possible approaches is a hub-and-spoke model of care which requires education and clear communication with primary care providers (Supplementary file 3) to optimize the quality of referrals. This approach could shift the initial work-up to primary care practices for many patients with cognitive symptoms, thereby potentially reducing wait times for specialty practices and decreasing the time to initiation of ATTs for appropriate patients.

In many health systems, neurology referrals are used for issues that arise commonly in dementia care, such as driving assessments, capacity concerns, and neuropsychiatric symptoms. For these patients, cognitive subspecialty clinics could consider utilizing advanced practice providers to expand capacity for physician specialists to initiate and manage ATTs. In larger centers with more resources, geriatric clinics or trained primary care practices may be most appropriate for the care of patients with advanced disease who may no longer be appropriate candidates for ATTs.

Neurology practices and cognitive subspecialty clinics may also consider establishing a fast-track pathway for potential ATT candidates. In this model, initial evaluations can be performed by advanced practice providers, nurses, particularly those with training as brain health navigators (15), or specially trained medical residents and fellows. Evaluation of fast-track patients should, at a minimum, include a qualifying cognitive assessment, data on risk factors for ARIA (e.g., uncontrolled hypertension, prior strokes, multiple microhemorrhages and superficial siderosis on MRI, anticoagulant use, and *APOE* status), general medical health, likelihood of needing future thrombolytic therapy, and contraindications to MRI use. More clinically complex patients requiring additional work-up may not be appropriate for fast-track evaluation.

Prior to engaging in shared decision-making with patients and their care partners, all providers should carefully read the relevant product labels (16, 17) and appropriate use recommendations for ATTs (18, 19) to be aware of the content and occasional differences between labeling and use recommendations. In addition to being medically appropriate, patients and their care partners should show interest in treatment and be able to reliably travel to multiple infusion, MRI, and clinic visits. Involvement of a social worker or other support services may be helpful.

Current care models aim to identify patients who are likely to achieve a clinically meaningful benefit with an ATT. We recommend collaboration with other providers when the benefit–risk profile is unclear. These include cases in which vascular, Lewy body, or other co-pathologies are suspected or in patients with atypical AD phenotypes (e.g., logopenic variant primary progressive aphasia, posterior cortical atrophy) where traditional screening tools can be less reliable. In large practices and academic centers, multidisciplinary review boards can review eligibility and may also facilitate coordination of care between prescribing physicians and infusion sites. Review board meetings should be held frequently and consistently to minimize delays. In smaller practices, we recommend a collaborative review of patient records and scans with another clinician to ensure that all relevant data have been considered.

Diagnostic tests are key to implementing ATTs. Providers should consider the accessibility of tests, potential financial and logistical burden on patients, reimbursement, and the availability of results at baseline and longitudinal time points. Testing should include a biomarker of amyloid pathology, *APOE* ε4 genotyping, and MRI with susceptibility-weighted imaging (SWI) or gradient echo (GRE) sequences to assess for pre-existing microhemorrhage or siderosis. Wherever possible, we recommend using the same MRI and PET scanner, MRI sequence (SWI vs. GRE), and PET tracer for baseline and follow-up scans. Artificial intelligence–based assistive software (e.g., Cortechs.ai, icometrix) may improve ARIA detection and diagnosis in the future (20). Radiology protocols for the detection and reporting of ARIA are available through the American Society of Neuroradiology (21).

Blood biomarkers (BBMs) may be used for disease confirmation in specialized memory-care practices or as part of initial assessment in broader settings, provided tests have ≥90% sensitivity and specificity (22). There remains practice variation on whether BBMs may serve as biomarkers of amyloid pathology for ATT eligibility decisions. Amyloid PET is the most well-established method for diagnosing amyloid pathology in symptomatic patients and is helpful for determining whether patients have cleared amyloid plaque sufficiently with therapy (16, 23). If amyloid PET is utilized for therapy eligibility or monitoring decisions, we recommend visual amyloid PET reads by expert interpreters, preferably augmented with Centiloid quantification (23). Although follow-up scans are typically scheduled 12 to 18 months after treatment initiation (19), lower baseline Centiloid values are associated with a shorter time to clearance (24, 25). This may justify an earlier amyloid PET scan in some patients for the purposes of treatment monitoring.

Reliable functional assessment is essential, because the distinctions between MCI, mild dementia, and moderate dementia—the boundary that largely determines candidacy—depends on the degree of functional impairment. Practices should use and document at least one validated instrument, such as the Clinical Dementia Rating (CDR), the Functional Activities Questionnaire (FAQ), the Alzheimer’s Disease Cooperative Study–Activities of Daily Living Inventory (ADCS-ADL), or the Function Assessment Tool (FAST), or carefully review the instrumental and basic activities of daily living (ADLs) with the patient and informant at baseline and follow-up to support both eligibility determination and longitudinal monitoring. Because functional trajectory is often the outcome of greatest importance to patients and care partners, consistent functional measurement may also inform ongoing decisions about continuation of therapy.

## 5 Patient management and monitoring

Additional clinic time with patients and their care partners is often needed prior to treatment initiation to review risks and benefits, address any residual concerns, and reinforce the importance of adhering to safety and care milestones. Cognitive specialists should facilitate shared clinical decision-making to ensure alignment of treatment goals and appropriate patient expectations. Patient education should provide a clear, relevant, and balanced perspective of the possible risks and benefits for each available treatment, as well as a discussion of *APOE* ε4 status to inform ARIA risk and potential familial implications. Discussions should establish expectations and rationales for treatment duration and acknowledge the dynamic nature of current research. Cognitive specialists may consider documenting the treatment plan with a signed agreement to ensure clear communication with patients and their care partners (Supplementary file 4).

Patient and care partner education should explain the signs and symptoms of ARIA, when it is more likely to occur, and when to call for guidance, visit the emergency department, or call 911. In addition to an FDA-recommended pocket medication card, patients should consider wearing a MedicAlert bracelet (or other similar identification method) that states they are receiving an ATT and that thrombolytics should be avoided. Additional training or communication should be provided to the patient’s local emergency department to ensure that patients receiving ATT are appropriately triaged, and that MRI (not CT) is used to rule out ARIA and distinguish it from an ischemic stroke during an emergency. The prescribing specialist’s team should establish an on-call system for emergencies during non-business hours.

Finally, logistical considerations are essential to support a positive patient experience. When needed, social workers and care navigators can be leveraged to support communication with insurance, overcome psychosocial barriers, and address other needs such as cross-state collaboration (e.g., for patients who travel south for the winter). Practices may consider connecting patients and their care partners to decentralized resources, such as manufacturer-provided customer support and advocacy-facilitated care and support. Treatment-specific support groups may help patients and care partners stay updated and share experiences with others on a similar patient journey. Simply scheduling all MRIs at the baseline visit can help ensure access, recognizing that schedules may need to be adjusted in the future. Any potential travel or financial needs should be addressed prior to initiating treatment.

## 6 Future directions

As the purpose of this article is to provide tools to address the bottleneck of patients awaiting care, the issue remains how to identify patients who have not yet come to clinical attention with a cognitive complaint. The Medicare Annual Wellness Visit (AWV) is a logical but underused opportunity: cognitive status is frequently not assessed systematically during the AWV, and only a minority of beneficiaries receive a formal screening test (26). Systematic cognitive assessment at the AWV has been associated with timelier first diagnosis of MCI (27), and neurologists can encourage and support AWV-based screening within their referring primary care networks. Health systems with sufficient informatics infrastructure may also consider automated electronic health record (EHR) phenotyping—combining structured data such as diagnostic codes and medications with information extracted from clinical notes—to generate outreach lists for proactive assessment; such models have demonstrated high discrimination for identifying MCI or AD and related dementias (28), and analyses of longitudinal clinical notes suggest cognitive signs are documented years before a formal diagnosis is made (29). Deployment of these tools requires appropriate institutional governance and local validation.

As high-performing assays such as plasma P-tau217 become more widely available, there is growing interest in their potential role in earlier case detection, including among primary care patients with cognitive concerns. The Alzheimer’s Association 2025 clinical practice guideline currently recommends blood-based biomarker use within specialized care settings (20); we note this boundary explicitly while recognizing that guidance in this area continues to evolve rapidly.

## 7 Conclusion

For the first time in more than 100 years since Alois Alzheimer characterized the disease bearing his name, we have treatments that can delay the associated cognitive and functional decline of this ultimately fatal disease. To implement these treatments, however, many systems, protocols, and procedures must be developed and coordinated across locations and clinicians within or between healthcare systems and practices. This coordination is achievable, but must emphasize timely diagnosis (including cognitive assessment, functional assessment, and biomarker confirmation of amyloid pathology) and initiation of treatment, since these are important factors in improving clinical outcomes (1–3, 8). A tracking system for infusions, MRI monitoring for ARIA, and (when appropriate) amyloid PET imaging is also key. Lastly, education for patients, care partners, and clinicians is essential. These elements can help enable the appropriate implementation of ATTs with optimized risks and maximal benefits.

## Data Availability

The original contributions presented in the study are included in the article/Supplementary material, further inquiries can be directed to the corresponding author.

## Footnotes

1
https://kisunla.lilly.com/hcp

2
https://www.leqembihcp.com/
