# Supplementary material for: Considerations for using amyloid-targeting therapies for the treatment of early symptomatic Alzheimer’s disease
Source: Front Neurol. 2026 Aug 10;17:1863897. doi: 10.3389/fneur.2026.1863897 (PMC13504022; doi:10.3389/fneur.2026.1863897)
Supplement: Supplementary file 1 [file Data_sheet_1.pdf]

Patient Name: ____

Date of Birth: ____

This form records our discussion of the potential benefits, risks, and alternatives to amyloid-targeting therapy (ATT), and confirms the monitoring and treatment plan we agreed upon. This form is not an informed consent for research or a waiver of any rights.

______ I hereby authorize infusion treatment with [insert drug name] to be performed by the staff at [insert practice name].

______ The potential risks and benefits associated with [insert drug name] have been explained to me including the possibility of amyloid-related imaging abnormalities (ARIA - swelling and bleeding in the brain).

______ I am aware infusion reactions may occur with [insert drug name], and I agree to emergency treatment if required.

______ The potential risks and benefits associated with available alternative treatments or taking no action have been explained to me.

______ I have informed my physician of any known allergies and of all the current medications and supplements I am taking.

______ I understand my genetic status (APOE testing) could increase my risk of developing ARIA, including symptomatic and serious ARIA.

______ I understand the importance of following the requirements of my course of therapy, including monitoring scans.

______ I have received a copy of the post-infusion instructions.

____

Signature of Patient/Care Partner   Date

____

Signature of Prescribing Physician   Date

This document should be reviewed and modified to meet practice-specific workflow needs and to preserve independent clinical judgment. The information herein does not supersede or replace current appropriate use recommendations or approved product labeling.
