# Supplementary material for: Considerations for using amyloid-targeting therapies for the treatment of early symptomatic Alzheimer’s disease
Source: Front Neurol. 2026 Aug 10;17:1863897. doi: 10.3389/fneur.2026.1863897 (PMC13504022; doi:10.3389/fneur.2026.1863897)
Supplement: Supplementary file 2 [file Data_sheet_2.pdf]

Letter to Primary Care Providers (template)
February 2026

Required Pre-Referral Information for Patients with Suspected Alzheimer's Disease

Dear Dr. Smith,

Thank you for your partnership in providing high-quality care for our shared patients. To ensure timely and effective evaluation in our clinic, we will now prioritize patient referrals for suspected Alzheimer's disease that include the following information. Early intervention with disease-modifying therapy is important for patients diagnosed with Alzheimer's disease, and this information will help us reduce the time to diagnosis.

Required Pre-Referral Information – Suspected Alzheimer's Disease

Please include the following in your referral packet:

1. Cognitive screening results (≥1 of the following)
    - [preferred cognitive tests]
2. Basic laboratory workup
    - CBC, CMP, TSH, and Vitamin B12 (additional labs as clinically indicated)
3. Relevant imaging
    - Brain MRI with [SWI or GRE] (preferred) or head CT if MRI contraindicated (include report and images if available)
4. Medication and medical history

Optional but Helpful

- Blood biomarkers for Alzheimer's disease (e.g., plasma P-tau217 assay)
    - Note: a positive plasma P-tau217 assay must be accompanied by a cognitive assessment with evidence of impairment
- *APOE* gene testing
- Social and functional assessment
    - Living situation, caregiver support, and any recent changes in activities of daily living

If you have questions or need assistance with screening tools, please contact our office at [Phone/Email]. We appreciate your collaboration in improving patient care.

Sincerely,

[Neurologist Name, MD]
[Practice Name]
[Contact Information]

This document should be reviewed and modified to meet practice-specific workflow needs and to preserve independent clinical judgment. The information herein does not supersede or replace current appropriate use recommendations or approved product labeling.
