# Supplementary material for: Considerations for using amyloid-targeting therapies for the treatment of early symptomatic Alzheimer’s disease
Source: Front Neurol. 2026 Aug 10;17:1863897. doi: 10.3389/fneur.2026.1863897 (PMC13504022; doi:10.3389/fneur.2026.1863897)
Supplement: Supplementary file 3 [file Data_sheet_3.pdf]

# Infusion Protocol for Amyloid-Targeting Therapies (ATTs)

## Contents

Purpose ........ 2
Reference Information ........ 2
Criteria for Treatment ........ 2
Dosing Information ........ 3
  Donanemab ........ 3
  Lecanemab ........ 3
Preparation, Storage, and Administration ........ 4
  Donanemab ........ 4
  Lecanemab ........ 6
Adverse Events ........ 7
  Infusion-related Reactions ........ 7
  Amyloid-related Imaging Abnormalities ........ 8
References ........ 11

This document should be reviewed and modified to meet practice-specific workflow needs and to preserve independent clinical judgment. The information herein does not supersede or replace current appropriate use recommendations or approved product labeling.

# Purpose

To provide guidance for the safe and effective administration of amyloid-targeting therapies (ATTs) for Alzheimer's disease to appropriate patients with accurate monitoring. Before starting, verify the information below with the approved product labeling.

# Reference Information

- For detailed information related to **donanemab**, please refer to the approved product labeling: https://kisunla.lilly.com/hcp

- For detailed information related to **lecanemab**, please refer to the approved product labeling: https://www.leqembihcp.com/

  NOTE: lecanemab is also available as a subcutaneous injection for maintenance after 18 months of treatment. Please refer to the approved product labeling for details regarding the subcutaneous injection.

# Criteria for Treatment

1. Prescriber is experienced in treating cognitive disorders.
2. Patient has a signed record of shared decision-making on file.
3. Patient meets criteria for Alzheimer's disease in the mild cognitive impairment (MCI) or mild dementia stage of disease.
4. Patient has documented confirmation of β-amyloid pathology.
5. Patient has completed *APOE* genotyping. The risk of ARIA across genotypes and the implications of testing results have been discussed with the patient.
6. Patient has had an MRI scan within the last 12 months.
7. Patient has no contraindications to receiving the prescribed treatment.
8. Prescriber has completed a clinical assessment of the risk for ARIA.
9. Neuroradiology is available to review serial MRI scans.
10. A process is in place to ensure the provider and pharmacy are notified to hold the infusion if the ordering provider has concerns or has not yet assessed patient data.

This document should be reviewed and modified to meet practice-specific workflow needs and to preserve independent clinical judgment. The information herein does not supersede or replace current appropriate use recommendations or approved product labeling.

# Dosing Information

## Donanemab

1. DOSE
    a. Dose 1: 350 mg
    b. Dose 2: 700 mg
    c. Dose 3: 1050 mg
    d. Dose 4 and beyond: 1400 mg
2. ROUTE OF ADMINISTRATION
    a. Intravenous infusion
3. RATE OF ADMINISTRATION
    a. Infuse dose over approximately 30 minutes
4. FREQUENCY OF ADMINISTRATION
    a. Infuse one dose every four weeks
    b. If an infusion is missed, resume administration every 4 weeks at the same dose as soon as possible
5. AVAILABLE AS
    a. Each vial contains 350 mg of donanemab (350 mg/20 mL)
    b. Donanemab is a clear to opalescent, colorless to slightly yellow to slightly brown solution in a single-dose vial
6. MEDICATIONS FOR POTENTIAL INFUSION-RELATED REACTIONS
    a. Orders for antihistamines, acetaminophen, and corticosteroids must be available for as needed use with each infusion

## Lecanemab

1. DOSE
    a. The recommended intravenous infusion dosage of lecanemab is 10 mg/kg that must be diluted
    b. Calculate the dose (mg), the total volume (mL) of lecanemab solution required, and the number of vials needed based on the patient's actual body weight and the recommended dose of 10 mg/kg
    c. Check the patients' weight at each infusion and document in kilograms
2. ROUTE OF ADMINISTRATION
    a. Intravenous infusion
3. RATE OF ADMINISTRATION
    a. Infuse dose over approximately one hour
4. FREQUENCY OF ADMINISTRATION

   a. Infuse one dose every two weeks
   b. If an infusion is missed, administer the next dose as soon as possible
5. AVAILABLE AS
   a. Each vial contains a lecanemab concentration of 100 mg/mL
   b. Lecanemab is a clear to opalescent and colorless to pale yellow solution, available as:
      i. Injection: 500 mg/5 mL (100 mg/mL) in a single-dose vial
      ii. Injection: 200 mg/2 mL (100 mg/mL) in a single-dose vial
6. MEDICATIONS FOR POTENTIAL INFUSION-RELATED REACTIONS
   a. Orders for antihistamines, acetaminophen, nonsteroidal anti-inflammatory drugs, or corticosteroids must be available for as needed use with each infusion

# Preparation, Storage, and Administration

## Donanemab

1. DILUTION
   a. Prior to administration, donanemab must be diluted with 0.9% sodium chloride injection
   b. Use aseptic technique when preparing the diluted donanemab solution for intravenous infusion
   c. Allow donanemab to equilibrate to room temperature before preparation
   d. Parenteral drug products should be inspected visually for particulate matter and discoloration prior to administration, whenever solution and container permit. Donanemab solution is clear to opalescent, colorless to slightly yellow to slightly brown. Do not use if particulate matter or discolorations are present
   e. Withdraw required volume of donanemab and mix with 0.9% sodium chloride injection, to the recommended total volume for a final concentration of 4 mg/mL to 10 mg/mL (see Table 4 from package labeling). Use only 0.9% sodium chloride injection for dilution
   f. Each vial is for one-time use only. Discard any unused portion left in the vial
   g. Gently invert the diluted donanemab solution to mix completely. Do not shake
   h. After dilution, immediate use is recommended. If the diluted donanemab solution is not administered immediately, store refrigerated at 2°C to 8°C

(36°F to 46°F) for up to 72 hours or at room temperature (20°C to 25°C [68°F to 77°F]) for up to 12 hours

  i. Do not freeze the diluted solution
  j. Storage times include the duration of infusion

2. STORAGE
   a. Store unopened vials refrigerated at 2°C to 8°C (36°F to 46°F)
   b. Keep the vial in the outer carton to protect from light
   c. If refrigeration is not available, may be stored at room temperature (20°C to 25°C [68°F to 77°F]) for up to 3 days
   d. After dilution, immediate use is recommended, if not administered immediately:
      i. Store refrigerated at 2°C to 8°C (36°F to 46°F) for up to 72 hours
      ii. Store at room temperature up to 20°C to 25°C [68°F to 77°F] for up to 12 hours
   e. Do not freeze or shake
3. ADMINISTRATION
   a. Confirm and document that an MRI was obtained prior to 2nd, 3rd, 4th, and 7th infusions
   b. Visually inspect the diluted donanemab solution for particles or discoloration prior to administration. Do not use if it is discolored, or opaque or foreign particles are seen
   c. Prior to infusion, if the diluted solution has been stored under refrigeration, allow the diluted donanemab solution to warm to room temperature
   d. Administer the entire diluted solution intravenously over approximately 30 minutes
   e. Promptly discontinue the infusion upon the first observation of any signs or symptoms consistent with a hypersensitivity-type reaction and contact the on-call neurologist
   f. Flush the line only with 0.9% sodium chloride injection at the end of the infusion per access specific line maintenance protocol
   g. Observe the patient post-infusion for a minimum of 30 minutes, and consider longer periods of observation if clinically indicated, to evaluate for infusion reactions and hypersensitivity reactions. The infusion rate may be reduced, or the infusion may be discontinued, and appropriate therapy administered as clinically indicated. Consider pre-medication at subsequent dosing with antihistamines, non-steroidal anti-inflammatory drugs, or corticosteroids

This document should be reviewed and modified to meet practice-specific workflow needs and to preserve independent clinical judgment. The information herein does not supersede or replace current appropriate use recommendations or approved product labeling.

## Lecanemab

1. DILUTION
    a. Prior to administration, lecanemab must be diluted in 250 mL of 0.9% sodium chloride injection, USP
    b. Use aseptic technique when preparing the lecanemab diluted solution for intravenous infusion
    c. Parenteral drug products should be inspected visually for particulate matter and discoloration prior to administration, whenever solution and container permit. Check that the lecanemab solution is clear to opalescent and colorless to pale yellow. Do not use if opaque particles, discoloration, or other foreign particles are present
    d. Remove the flip-off cap from the vial. Insert the sterile syringe needle into the vial through the center of the rubber stopper
    e. Withdraw the required volume of lecanemab from the vial(s) and add to an infusion bag containing 250 mL of 0.9% sodium chloride injection, USP
    f. Each vial is for one time-use only. Discard any unused portion
    g. Gently invert the infusion bag containing the lecanemab diluted solution to mix completely. Do not shake
2. STORAGE
    a. Immediate use is recommended
    b. If not administered immediately:
        i. Store refrigerated at 2°C to 8°C (36°F to 46°F) for up to 4 hours
        ii. Store at room temperature up to 30°C (86°F) for up to 4 hours
    c. Do not freeze
3. ADMINISTRATION
    a. Confirm and document that an MRI was obtained prior to the 3rd, 5th, 7th, and 14th infusions
    b. Visually inspect the lecanemab diluted solution for particles or discoloration prior to administration. Do not use if it is discolored, or opaque or foreign particles are seen
    c. Prior to infusion, allow the lecanemab diluted solution to warm to room temperature
    d. Infuse the entire volume of the lecanemab diluted solution intravenously over approximately one hour through an intravenous line containing a terminal low-protein binding 0.2 micron in-line filter. Flush infusion line to ensure all lecanemab is administered

This document should be reviewed and modified to meet practice-specific workflow needs and to preserve independent clinical judgment. The information herein does not supersede or replace current appropriate use recommendations or approved product labeling.

e. Monitor for any signs or symptoms of an infusion-related reaction during the infusion and consider longer periods of observation if clinically indicated. The infusion rate may be reduced, or the infusion may be discontinued, and appropriate therapy administered as clinically indicated. Consider pre-medication at subsequent dosing with antihistamines, non-steroidal anti-inflammatory drugs, or corticosteroids

# Adverse Events

## Infusion-related Reactions

1. SIGNS AND SYMPTOMS
    a. Donanemab: Chills, erythema, nausea/vomiting, flushing, difficulty breathing/dyspnea, sweating, elevated blood pressure, headache, chest pain, and low blood pressure
    b. Lecanemab: Fever and flu-like symptoms (chills, generalized aches, feeling shaky, and joint pain), nausea, vomiting, hypotension, hypertension, and oxygen desaturation
2. PREVENTION
    a. Prophylactic treatment with antihistamines, acetaminophen, nonsteroidal anti-inflammatory drugs, or corticosteroids prior to future infusions may be considered
3. TREATMENT
    a. In the event of an infusion-related reaction, the infusion rate may be reduced, or the infusion may be discontinued, and appropriate therapy initiated as clinically indicated
        i. Hold infusion and page the on-call provider
        ii. Rescue medications available for as needed symptoms of infusion-related reaction:
            1. diphenhydramine 25 mg IV once
            2. hydrocortisone 100 mg IV once
            3. acetaminophen 650 mg orally once
        iii. Monitor for symptom resolution, repeat as directed by provider
        iv. Determine a pre-medication strategy for subsequent infusions
        v. Rescue medications must be available with each infusion
4. MONITORING
    a. Patients should be advised to stay for post-infusion for monitoring of infusion related reactions. The duration of monitoring may vary depending on the

This document should be reviewed and modified to meet practice-specific workflow needs and to preserve independent clinical judgment. The information herein does not supersede or replace current appropriate use recommendations or approved product labeling.

specific ATT, the number of prior infusions, and the patient's experience with previous infusions. For ATT-specific guidance, please refer to the most current appropriate use recommendations for donanemab [1] and lecanemab [2].

## Amyloid-related Imaging Abnormalities

1. OVERVIEW
    a. Monoclonal antibodies directed against aggregated forms of beta amyloid, including donanemab and lecanemab, can cause amyloid related imaging abnormalities (ARIA), characterized as ARIA with edema (ARIA-E), which can be observed on MRI as brain edema or sulcal effusions, and ARIA with hemosiderin deposition (ARIA-H), which includes microhemorrhage and superficial siderosis
    b. ARIA can occur spontaneously in patients with Alzheimer's disease, particularly in patients with MRI findings suggestive of cerebral amyloid angiopathy, such as pretreatment microhemorrhage or superficial siderosis. ARIA-H associated with monoclonal antibodies directed against aggregated forms of beta amyloid generally occurs in association with an occurrence of ARIA-E. ARIA-H of any cause and ARIA-E can occur together
2. SYMPTOMS
    a. ARIA usually occurs early in treatment and is usually asymptomatic, although serious and life-threatening events, including seizure and status epilepticus, can occur. ARIA can be fatal. When present, reported symptoms associated with ARIA may include headache, confusion, visual changes, dizziness, nausea, and gait difficulty. Focal neurologic deficits may also occur. Symptoms associated with ARIA usually resolve over time
    b. In addition to ARIA, intracerebral hemorrhages greater than 1 cm in diameter have occurred in patients treated with ATTs
3. MONITORING
    a. Obtain a recent brain magnetic resonance imaging (MRI) prior to initiating treatment with lecanemab or donanemab
    b. Obtain an MRI for:
        i. Lecanemab prior to the 3$^{rd}$, 5$^{th}$, 7$^{th}$, and 14$^{th}$ infusions
        ii. Donanemab prior to the 2$^{nd}$, 3$^{rd}$, 4$^{th}$ and 7$^{th}$ infusions
    c. Enhanced clinical vigilance for ARIA is recommended during the first 14 weeks of lecanemab treatment and for the first 24 weeks of donanemab

treatment. If a patient experiences symptoms suggestive of ARIA, clinical evaluation should be performed, including MRI if indicated

4. DOSING ADJUSTMENTS
    a. In patients who develop intracerebral hemorrhage greater than 1 cm in diameter during treatment with lecanemab or donanemab, suspend dosing until MRI demonstrates radiographic stabilization and symptoms, if present, resolve. Use clinical judgement in considering whether to continue treatment after radiographic stabilization and resolution of symptoms or permanently discontinue treatment

This document should be reviewed and modified to meet practice-specific workflow needs and to preserve independent clinical judgment. The information herein does not supersede or replace current appropriate use recommendations or approved product labeling.

**Table 1: Dosing Recommendations for Patients with ARIA-edema which can be observed on MRI as brain edema or sulcal effusions**

<table>
<tr><th rowspan="2">Clinical Symptom Severity[1]</th><th colspan="3">ARIA-E Severity on MRI</th></tr>
<tr><th>Mild</th><th>Moderate</th><th>Severe</th></tr>
<tr><td>Asymptomatic</td><td>May continue dosing</td><td>Suspend dosing[2]</td><td rowspan="3">Suspend dosing[2]</td></tr>
<tr><td>Mild</td><td>May continue dosing based on clinical judgement</td><td>Suspend dosing[2]</td></tr>
<tr><td>Moderate or Severe</td><td colspan="2">Suspend dosing[2]</td></tr>
</table>

[1] Clinical Symptom Severity Categories:

Mild: discomfort noticed, but no disruption of normal daily activity.

Moderate: discomfort sufficient to reduce or affect normal daily activity.

Severe: incapacitating, with inability to work or to perform normal daily activity.

[2] Suspend until MRI demonstrates radiographic resolution and symptoms, if present, resolve; consider a follow-up MRI to assess for resolution 2 to 4 months after initial identification. Resumption of dosing should be guided by clinical judgment.

**Table 2: Dosing Recommendations for Patients with ARIA-hemosiderin deposition which includes microhemorrhage and superficial siderosis**

<table>
<tr><th rowspan="2">Clinical Symptom Severity</th><th colspan="3">ARIA-H Severity on MRI</th></tr>
<tr><th>Mild</th><th>Moderate</th><th>Severe</th></tr>
<tr><td>Asymptomatic</td><td>May continue dosing</td><td>Suspend dosing[1]</td><td rowspan="2">Suspend dosing[2]</td></tr>
<tr><td>Symptomatic</td><td>Suspend dosing[1]</td><td>Suspend dosing[1]</td></tr>
</table>

[1] Suspend until MRI demonstrates radiographic stabilization and symptoms, if present, resolve; resumption of dosing should be guided by clinical judgment; consider a follow-up MRI to assess for stabilization 2 to 4 months after initial identification.

[2] Suspend until MRI demonstrates radiographic stabilization and symptoms, if present, resolve; use clinical judgment in considering whether to continue treatment or permanently discontinue.

This document should be reviewed and modified to meet practice-specific workflow needs and to preserve independent clinical judgment. The information herein does not supersede or replace current appropriate use recommendations or approved product labeling.

# References

1. Rabinovici GD, Selkoe DJ, Schindler SE, et al. Donanemab: Appropriate use recommendations. J Prev Alzheimers Dis 2025;12:100150. doi: 10.1016/j.tjpad.2025.100150
2. Cummings J, Apostolova L, Rabinovici GD, et al. Lecanemab: Appropriate use recommendations. J Prev Alzheimers Dis 2023;10:362-77. doi: 10.14283/jpad.2023.30
