# Supplementary material for: Considerations for using amyloid-targeting therapies for the treatment of early symptomatic Alzheimer’s disease
Source: Front Neurol. 2026 Aug 10;17:1863897. doi: 10.3389/fneur.2026.1863897 (PMC13504022; doi:10.3389/fneur.2026.1863897)
Supplement: Supplementary file 4 [file Data_sheet_4.pdf]

Patient Details

Patient Name: ____ DOB: __________________

MRN: ____________________ Insurance: _____________________ Medicare #: ___________________

Primary Contact Name: _____________________________ Preferred Phone Number: __________________

Referring Physician: _________________________________ Referral date: ________________________

Referral reason: ____

Eligibility Review

	Date Reviewed	Notes
**Relevant medical history**		
**Relevant social history**		
**Medications**		
**Physical/neurological exam**		
**Cognitive assessment**		
**Functional assessment**		
**Laboratory results**		
**Blood pressure review**		
**Anticoagulant treatment**		
**Blood biomarkers**		
APOE **genetic testing**		
**Brain MRI**		
**Amyloid PET scan**		
**Access to reliable transportation**		
**Care partner support**		

This document should be reviewed and modified to meet practice-specific workflow needs and to preserve independent clinical judgment. The information herein does not supersede or replace current appropriate use recommendations or approved product labeling.

Treatment Decision and Preparation

	Date Completed	Notes
**Full eligibility confirmation**		
**Patient/care partner treatment discussion**		
**Patient/care partner education and handouts**		
**Shared decision-making documentation**		
**Patient's primary care provider updated**		
**Patient's medication list updated**		
**Enrollment in manufacturer's patient support program**		
**Insurance authorization**		
**Patient added to applicable treatment registry**		

Treatment Plan

Diagnosis: ____

Treatment Plan: ____

____

____

Infusion location and details: ____

____

Notes: ____

____

____

This document should be reviewed and modified to meet practice-specific workflow needs and to preserve independent clinical judgment. The information herein does not supersede or replace current appropriate use recommendations or approved product labeling.

Donanemab Infusion Tracking

	Anticipated Date	Actual Date	Notes
**Infusion #1**			
**MRI**			
**Infusion #2**			
**MRI**			
**Infusion #3**			
**MRI**			
**Infusion #4**			
**Infusion #5**			
**Infusion #6**			
**MRI**			
**6-month appointment**			• Cognitive/functional assessment<br>• Update applicable registry
**Infusion #7**			
**Infusion #8**			
**Infusion #9**			
**Infusion #10**			
**Infusion #11**			
**Infusion #12**			
**12-month appointment***			• Cognitive/functional assessment<br>• Update applicable registry
**Additional Testing**			
**Amyloid PET**			
**Blood biomarkers**			
**Additional MRI**			

Note: After each infusion, contact the patient to monitor symptoms of infusion-related reactions or new neurologic symptoms.

*Continue or discontinue treatment as indicated by the product label and appropriate use recommendations. After treatment completion, discuss the appropriate treatment plan.

This document should be reviewed and modified to meet practice-specific workflow needs and to preserve independent clinical judgment. The information herein does not supersede or replace current appropriate use recommendations or approved product labeling.

<u>Lecanemab Infusion Tracking</u>

	Anticipated Date	Actual Date	Notes
**Infusion #1**			
**Infusion #2**			
**MRI**			
**Infusion #3**			
**Infusion #4**			
**MRI**			
**Infusion #5**			
**Infusion #6**			
**MRI**			
**6-month appointment**			• Cognitive/functional assessment<br>• Update applicable registry
**Infusion #7**			
**Infusion #8**			
**Infusion #9**			
**Infusion #10**			
**Infusion #11**			
**Infusion #12**			
**Infusion #13**			
**MRI**			
**12-month appointment**			• Cognitive/functional assessment<br>• Update applicable registry
**Infusion #14**			
**Infusion #15**			
**Infusion #16**			
**Infusion #17**			
**Infusion #18**			
**Infusion #19**			
**Infusion #20**			
**Infusion #21**			

This document should be reviewed and modified to meet practice-specific workflow needs and to preserve independent clinical judgment. The information herein does not supersede or replace current appropriate use recommendations or approved product labeling.

**Infusion #22**			
**Infusion #23**			
**Infusion #24**			
**18-month appointment***			• Cognitive/functional assessment<br>• Update applicable registry
**Additional Testing**			
**Amyloid PET**			
**Blood biomarkers**			
**Additional MRI**			

Note: After each infusion, contact the patient to monitor symptoms of infusion-related reactions or new neurologic symptoms.

*Continue or discontinue treatment as indicated by the product label and appropriate use recommendations. After treatment completion, discuss the appropriate treatment plan.
